# The impact of loco-regional treatment on ipsilateral breast cancer recurrence and outcomes in carriers of *BRCA1/2* pathogenic variants

**DOI:** 10.1007/s10549-025-07658-x

**Published:** 2025-03-05

**Authors:** Rinat Bernstein-Molho, Ory Haisraely, Shira Galper, Narmeen Abu-Shhada, Einav Nili Gal-Yam, Tehillah S. Menes, Philip Poortmans, Orit Kaidar-Person

**Affiliations:** 1 The Suzanne Levy-Gertner Oncogenetics Unit, Breast Cancer Center, Oncology Institute, Sheba Tel Hashomer Medical Center, Tel- Hashomer, 52621 Ramat-Gan, Israel; 2 Breast Cancer Institute, The Jusidman Cancer Center, Chaim Sheba Tel Hashomer Medical Center, Ramat-Gan, Israel; 3 Breast Radiation Unit, The Jusidman Cancer Center, Chaim Sheba Tel Hashomer Medical Center, Ramat-Gan, Israel; 4 Department of Surgery, Chaim Sheba Tel Hashomer Medical Center, Ramat-Gan, Israel; 5 School of Medicine, Faculty of Medical and Health Sciences, Tel-Aviv University, Tel-Aviv, Israel; 6 Department of Radiation Oncology, Iridium Netwerk, Wilrijk-Antwerp, Belgium; 7 Faculty of Medicine and Health Sciences, University of Antwerp, Wilrijk-Antwerp, Belgium; 8 GROW-School for Oncology and Developmental Biology, Maastricht University, Maastricht, The Netherlands

**Keywords:** *BRCA1/2* germline pathogenic variants, Mastectomy, Radiation, Ipsilateral breast tumor recurrence, Breast cancer

## Abstract

**Purpose:**

Our previous data showed that carriers of germline *BRCA1/2* pathogenic variants (PV) with breast cancer (BC) treated with mastectomy without post-mastectomy radiation therapy (PMRT) had higher rates of loco-regional recurrence (LRR) compared to those who underwent PMRT or breast-conserving therapy (BCT), despite earlier stage BC. Our aim was to verify our previous findings in a larger cohort.

**Methods:**

Clinical data were extracted from the medical records of *BRCA1/2* mutation carriers with BC, treated at a single institution between 1/2006 and12/2022. The data included demographics, treatment modalities, and BC outcomes.

**Results:**

A total of 464 patients with 484 primary tumors were analyzed. Of these, 48.3% mastectomies were performed: 66% (154) without PMRT (non-PMRT) and 34% (80) with PMRT; 51.8% (250) underwent BCT. The non-PMRT group had earlier disease stages at diagnosis (77.3% were Tis and T1N0 stage) compared to the PMRT and BCT groups (3.8% and 45%, respectively, p < 0.001). During the study period with a median follow-up time of 75 months (range 12–211), the LRR rate was 13% (20/154) in the non-PMRT cohort compared with 1.25% (1/80) in the PMRT group (p = 0.003), and 6.4% (16/250) in the BCT group (p = 0.03). Cumulative incidence of LRR at 5 and 15 years was 14.7%, and 16.6% in the non-PMRT, compared to 5.1% and 35% in the BCT group, respectively (p = 0.081). No significant difference in overall survival was observed (p = 0.202).

**Conclusions:**

The timing and rates of LRRs differ according to the loco-regional therapy, which might indicate a different etiology driving these events.

## Introduction

Women with germline pathogenic variants (PVs) in *BRCA1* or *BRCA2* genes have a significantly increased lifetime risk for breast cancer (BC) compared with the general population [1]. The estimated overall lifetime risk of breast cancer in *BRCA1/2* PV carriers ranges between 50 and 80%. Therefore, these patients are offered bilateral risk-reducing mastectomy, and in the case of breast cancer, therapeutic mastectomy, and contralateral risk-reducing mastectomy are discussed [2, 3]. Studies in *BRCA1/2* PV carriers are conflicting regarding the equivalence of breast-conserving therapy (breast-conserving surgery and radiation therapy, BCT) to mastectomy concerning local failure rates [4–8], while survival is similar [8–12]. Therefore, the choice of surgical procedure to treat primary breast cancer, considering ipsilateral breast tumor recurrence (IBTR) risk and the risk of contralateral disease, should be a shared decision with the patient. This should be based on multiple factors like age, family history (when informative), overall prognosis from the current or previous cancer, ability of the patient to undergo appropriate surveillance, comorbidities, life expectancy, as well as other non-surgical options to reduce the risk for breast cancer in the contralateral breast [13, 14].

Notably, none of the published reviews or meta-analyses [9–12] demonstrating higher risks of local failure in BCT vs mastectomy cohorts in *BRCA* PV carriers, considered post-mastectomy radiation therapy (PMRT) or avoidance of it as a risk factor for IBTR. Our previous data showed that carriers of *BRCA1/2* PVs with BC treated with mastectomy without PMRT had higher rates of loco-regional recurrence (LRR), occurring earlier after surgery compared to those who underwent PMRT or BCT, despite having an earlier BC stage [15]. As most of the mastectomy patients underwent skin-sparing or nipple-sparing mastectomy (SSM/NSM), we assumed that LRR could be the result of new cancer growth in the remaining breast tissue and/or a result of residual subclinical disease.

As our previous results were alerting and relevant for loco-regional management, our aim in this study was to validate our previous findings of local failure patterns in a larger cohort of *BRCA1/2* PV carriers undergoing different types of local treatment at primary diagnosis of BC, with longer follow-up.

## Methods

This study received ethical approval from Sheba Medical Center’s ethics committee. As part of the standard genetic counseling process, all patients provided informed consent for initial *BRCA1/2* genetic testing and data collection.

Prospectively maintained databases within the Sheba Medical Center’s Oncogenetics and Oncology Institute units were queried. The search focused on *BRCA1* and *BRCA2* pathogenic variant (PV) carriers diagnosed with breast cancer (BC) between January 2006 and December 2022. Clinical data including patient characteristics, genetic testing, breast cancer characteristics, treatment modalities (including systemic therapy and surgery), date and site of recurrence, and status at last follow-up were documented. Only patients with at least 6 months of clinical follow-up after BC diagnosis were included in the analysis. Systemic therapy was defined as chemotherapy administered with or without anti-HER2 therapy, excluding endocrine therapy.

The non-PMRT group included patients who underwent mastectomy as their primary surgical treatment without receiving PMRT, and patients who opted for mastectomy following a positive genetic test result after initial breast-conserving surgery without radiation.

### Statistical methods

All variables were tabulated by descriptive statistics.

The chi-square test was applied to test the difference in categorical variable distribution between treatment groups. The two-sample T-test was applied to test the difference in mean age between treatment groups. Mann–Whitney and Kruskal–Wallis test were used for non-parametric continuous/ordinal variables. Survival analysis was applied to test the statistical significance of the survival difference between treatment groups using the Kaplan–Meier survival function curve. The hazard ratio for survival between the treatment groups was estimated via the Cox regression model.

Our endpoint is LRR as the first failure, including all breast/chest wall invasive recurrences and recurrences in the regional lymph nodes (axilla levels 1–4, internal mammary), without concurrent distant failure. After interactions and co-linearity examinations, we analyzed hazard ratio calculations for time to LRR as the first event via the multivariable Cox regression model for BCT and non-PMRT groups, between age groups, *BRCA* gene PVs, between tumor size (T), lymph node stage (N), HER2 expression, Estrogen receptor (ER) expression, systemic therapy (chemotherapy with/without anti-HER2), and type of surgery. The time-to-event endpoints were censored at the time of competing events, if present. Distant recurrence, non-breast cancer, contralateral BC (CBC) with treatment including systemic therapy, mastectomy without LRR in women who initially underwent BCT, and death were considered competing events for LRR.

All tests were two-tailed, and a p-value of 5% or less was considered statistically significant. The data was analyzed using the SPSS ® version 29 (IBM company, Chicago, OH).

## Results

Overall, 481 BC patients with *BRCA1/2* germline PVs were identified, and 20 of them presented with synchronous bilateral disease. The median follow-up time was 75.3 months (range 12.5–211). After excluding 16 patients with metastatic de novo disease and 1 patient not operated for medical reasons, 464 patients with 484 primary tumors were analyzed. Table 1 presents the relevant clinical features of study participants. Compared to other groups, in the non-PMRT group, there were more patients with *BRCA1* PVs and a higher proportion of patients with a knowledge of their genetic status before BC diagnosis. However, these facts were not reflected in either the mean age at diagnosis or the percentage of TN tumors.

**Table 1:** Patient characteristics by treatment group

	Mastectomy with RT	Mastectomy without RT	*p*-value^#^	BCT	*p*-value^##^
Number of cases (20 pts with bilateral disease)	80 (6 bilateral)	154 (8 bilateral)		250 (6 bilateral)	
Median follow-up, months (range)	83.6 (13–188)	81.3 (12–195)		70.6 (11.5–211)	
Mean age at diagnosis, years (range)	45.6 (25–79)	44.5 (27–67)	0.42	45.4 (24–80)	0.43
Age distribution: ≥ 40 years old, No. (%)	36 (48.6)	79 (54.1)	0.29	171 (70)	< 0.001
Mutation in, No. (%)			< 0.001		< 0.001
*BRCA1*	45 (56.3)	120 (78)		152 (60)	
*BRCA2*	34 (42.5)	33 (21.4)		94 (37.6)	
*Both BRCA1* and* BRCA2*		1 (0.6)		4 (1.6)	
Unknown	1				
Awareness of genetic status before BC diagnosis	19/74 (26%)	96/154 (66%)	< 0.001	80/244 (33%)	< 0.001
Performed BSO prior to BC diagnosis, No. (%)	7/74 (9.5)	39/146 (26.7)	0.002	51/244 (20.9)	0.233
Stage at diagnosis ^a^					
DCIS, No. (%)	0	28 (18.2)	< 0.001	25 (10)	< 0.001
T1N0, No. (%)	3 (3.75)	91 (59.1)		89 (35.6)	
T2-3N0, No. (%)	7 (8.75)	24 (15.6)		42 (16.8)	
Node-positive disease, No. (%)	67 (83.75)	8 (5.2)		91 (36.4)	
Invasive disease cases^b^	80	125		225	
Triple-negative, No. (%)	40 (50)	77 (61.6)	0.14	128 (56.9)	0.506
ER-positive Her2-negative, No. (%)	35 (43.8)	38 (34.4)		82 (36.4)	
ER any Her2-positive, No. (%)	5 (6.3)	10 (8)		15 (6.7)	

*BC* Breast cancer; *BCT* breast conserving therapy; *BSO* bilateral salpingo-oophorectomy; *DCIS* ductal carcinoma in situ; *IBTR* ipsilateral breast tumor recurrence; *RT* radiation therapy

^#^Referring to Mastectomy with vs without RT

^##^Referring to BCT compared to Mastectomy without RT

^a^In patients with bilateral disease, each cancer is considered as a separate case, excluding patients with unknown T or N status (3 in each group);

^b^Excluding DCIS

### Surgery

All patients underwent breast MRI before surgery, following the institution's standard protocol.

In the non-PMRT group, 94.8% (146/154) of the patients had SSM or NSM, and 5.2% (8/154) had simple or modified radical mastectomy. In the PMRT cohort, 67.5% (54/80) had SSM or NSM, 31.3% (25/80) had simple or modified radical mastectomy, and in one patient information was unavailable.

In the subgroup of 57 patients who underwent mastectomy following lumpectomy without RT following positive *BRCA* testing (non-PMRT group), the median time between lumpectomy and mastectomy was 6.92 months (range 1.44–10.75).

All surgical margins of the final surgical procedure for the primary breast cancer were reported as negative.

### Radiation therapy

Not all patients were irradiated at our institution [15]. Radiation planning was based on 3D-planning, photon-based techniques. The total dose/fractionation was at the discretion of the treating radiation oncologist. Dose and fractionation varied, with most immediate reconstruction cases receiving 1.8–2 Gy per fraction to a total dose of 45–50.4 Gy and BCT or chest wall with/without regional nodes mostly 2.65–2.67 Gy per fraction to a total dose of 40.05–42.4 Gy. Boost irradiation, photon or electron-based, was delivered according to the physician’s preference.

### Systemic therapy

Significantly more patients received chemotherapy in the PMRT group (mostly preoperative) and BCT (mostly postoperative) compared with the non-PMRT cohort (*p* = 0.002) (Table 2). Patients with hormone receptors-positive tumors and Her2-positive tumors received hormonal therapy and anti-Her2 therapy according to established protocols.

**Table 2:** Surgical and oncological treatments by group

	PMRT	Non-PMRT	*p*-value^#^	BCT	*p*-value^##^
Number of cases	80	154		250	
Bilateral mastectomy at the time of primary surgery, No. (%)	48 (59.3)	91 (59)	P = 0.948		
Subsequent contralateral RRM, No. (%)	13 (16.25)	57 (37)	P < 0.001		
Subsequent bilateral mastectomy after BCT				42 (16.8)	
RRM				21	
Following IBTR				8	
Following CBC / synchronous bilateral disease				13	
Chemotherapy					
Overall, No. (%)	76 (95)	88 (57.1)	P < 0.001	179 (71.6)	P = 0.002
Post-operative	18/76 (23.7)	64/88 (72.7)	P < 0.001	97/179 (54.2)	P < 0.001
Pre-operative	58/76 (76.3)	24/88 (27.3)		82/179 (45.8)	
Refused recommended chemotherapy	0	5^a^		6^b^	

*BCT* breast conserving therapy; *CBC* contralateral breast cancer; *IBTR* ipsilateral breast tumor recurrence; *RT* radiation therapy; *RRM* risk reducing mastectomy; *PMRT* postmastectomy RT

^#^Referring to Mastectomy with vs without RT

^##^Referring to Lumpectomy vs Mastectomy without RT (non-PMRT)

^a^4/5 patients had triple-negative disease (3 with stage I, one with stage III), and 1 patient had T1bN0 ER-positive Her2-negative disease with Oncotype RS-22

^b^5/6 patients had T1N0 triple-negative disease (2 with T1bN0, 2 with T1cN0, one T1 not specified), 1/6 had hormone receptor-positive / Her2-negative T2N0 disease

### Patterns of recurrence

At a median follow-up of 75.3 months, LRR as *first* failure was more common in the non-PMRT group (13%; 20/154 breasts versus the PMRT group (1.25%; 1/80 breasts; p = 0.003), and the BCT group (6.4%; 16/250; p = 0.024) (Table 3). Twelve of the 20 (60%) recurrences in the non-PMRT group were in patients with T1N0 disease at initial presentation, and one invasive recurrence was in a patient with pure DCIS at presentation. Patterns of LRR in different subgroups are presented in Tables 3 and 4.

**Table 3:** Outcomes by group

	Mastectomy with RT	Mastectomy without RT	*p*-value^#^	Lumpectomy with RT	*p*-value^##^
Number of breasts (patients)*	80 (80)	154 (142)		250 (244)	
*Recurrent disease (first event), No*					
Ipsilateral recurrence (% of breasts)	1 (1.25)	20 (13)^c^	0.003	16 (6.4)^e^	0.03
Contralateral metachronous second primary (out of patients without RRM)^&^	3/24 (12.5%)^a^	0/7	NS	29/209 (13.9%)	NS
Contralateral metachronous second primary (out of patients after RRM)^&^	1/50 (2)	1/125 (0.8)	NS	0/29	NS
Distant recurrence (% of patients)	11/80 (13.8)	1/142 (0.7)	P < 0.001	22/244 (9)	P < 0.001
Non-BC after BC diagnosis (% of patients)	2/80 (2.5)^b^	6/142 (4.2)^d^	NS	5/244 (2)^f^	NS
*Patterns of ipsilateral recurrence*					
Breast / chest wall (% of breasts)	1	15 (9.7)		15	
Axillary LN (% of breasts)	0	4 (2.6)		1	
Both breast/chest wall and LN (% of breasts)	0	1 (0.6)		0	
*Status at last follow-up, No. (% of patients)*					
Alive (all)	71 (88.75)	134 (94.4)	P = 0.503	228 (93.4)	P = 0.22
Alive without disease	67 (83.75)	132 (93)		212 (86.9)	
Alive with disease	4 (5)	4 (2.8)		16 (6.6)	
Deceased (BC)	7 (8.75)	05 (3.5)		12 (4.9)	
Deceased (other causes)	1 (1.25)	0		3 (1.2)	

*BC* Breast cancer; *LN* lymph nodes; *NS* non-significant; *RT* radiation therapy

*For distant recurrences, non-breast cancer diagnosis, and status at follow-up, patients with synchronous bilateral disease at diagnosis were counted only in one group considering the more advanced tumor at presentation

^&^Excluding patients with bilateral disease at primary diagnosis

^#^Referring to Mastectomy with vs without RT

^##^Referring to Lumpectomy vs Mastectomy without RT

^a^One of the patients received prophylactic RT to contralateral breast at presentation as part of a trial

^b^1 patient had ovarian cancer two years after BC, 1 patient had bronchoalveolar lung carcinoma 4 months after breast cancer diagnosis

^c^4/19 patients had metastatic disease 20–37 months following IBTR

^d^2 patients with ovarian / primary peritoneal cancer, 1 with cervical cancer, 1 with endometrial cancer, 1 with neuroendocrine pancreatic cancer, 1 with melanoma

^e^3/16 had metastatic disease 16–28 months following IBTR

^f^2 patients with gastric cancer, 1 with pancreatic cancer, 1 with rectal cancer, 1 with B-cell lymphoma

**Table 4:** Characteristics and patterns of ipsilateral recurrence in patients undergoing mastectomy without RT

	Upfront mastectomy N = 97	Lumpectomy > mastectomy N = 57	*p*-value
DCIS	23 (23.7)	5 (8.8)	0.02
T1N0	49 (50.5)	39 (68.4)	0.03
Node-positive disease	6 (6.2)	2 (3.5)	0.47
Triple-negative disease	48 (49.5)	31 (54.4)	0.56
Chemotherapy ± anti-Her2 therapy	46 (47.4)	42 (73.7)	0.001
*Patterns of ipsilateral recurrence*			
Breast/chest wall (% of breasts)	13 (13.4)	2 (3.5)	0.046
Axillary LN (% of breasts)	1 (1)	3 (5.2)	0.11
Both breast/chest wall and LN (% of breasts)	1 (1)	0	0.44

*DCIS* ductal carcinoma in situ; *LN* lymph nodes; *RT* radiation therapy

**Fig. 1:**
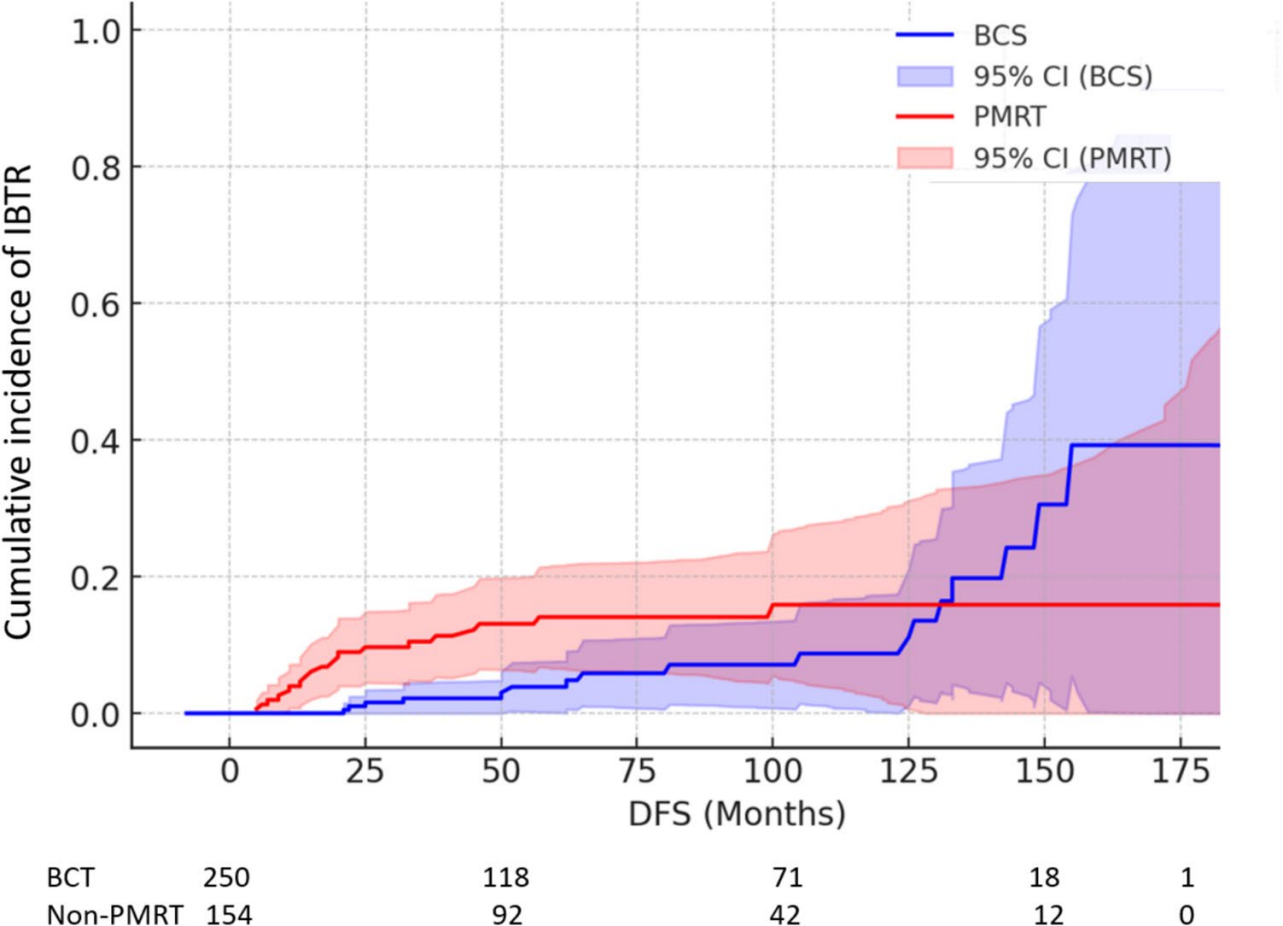
Cumulative incidence of ipsilateral breast tumor recurrence. *PMRT* post-mastectomy radiation therapy

Median time to LRR was 17.5 months (range 6–100) for non-PMRT patients and 69.9 months (range 16–153.7) for BCT patients. The mean time to LRR for patients in the BCT cohort was estimated to be 174.9 months (95% CI 161.5–188.4), while for non-PMRT patients, the mean time to LRR was 169.2 months (95% CI 158.7–179.7). In 80% (16/20) of the recurrences in the non-PMRT group, and in 87.5% (14/16) of the recurrences in the BCT group, the recurrent tumors exhibited the same receptor characteristics as the primary tumors. The time to the single LRR event in the PMRT group was 99 months. The recurrent tumor receptors were identical to the primary tumor. Figure 1 shows that at the time point of ~ 130 months of follow-up, the cumulative incidence of LRR in the BCT group increased and exceeded that of the non-PMRT group. Cumulative incidence rates at 5, 10, and 15 years were 14.7%, 16.6%, and 16.6% (95% confidence interval (CI) 11.43–20.57%) in the non-PMRT, compared to 5.1%, 10.3% and 35% (95% CI 30.01–41.99%) in BCT group, respectively (p = 0.081).

### Risk factors for IBTR

Univariate analysis to evaluate factors associated with IBTR in the non-PMRT or BCT cohorts (age < 40 vs > 40, *BRCA1* vs. *BRCA2*, T0-1 vs T2-4, N0 vs N1-3, chemotherapy vs no chemotherapy) did not show any risk factor significantly predicting IBTR, therefore no multivariate analysis was performed.

### Contralateral breast cancer

In the non-PMRT group, 96% of patients underwent contralateral RRM (at the time of therapeutic mastectomy or later), compared to 76.3% in the PMRT group (Table 2). In the BCT group, only 9.2% had contralateral RRM either at primary diagnosis or subsequently. There was no statistical difference in the incidence of CBC between the groups (Table 3).

Out of 204 patients who underwent RRM in the whole cohort, only 1 (0.5%) patient presented with a triple-negative T1N2 CBC, 11 years after RRM.

### Salvage treatments after loco-regional recurrence

Following LRR in the non-PMRT group, pre- or postoperative chemotherapy was administered to 50% (10/20) of patients. In half of the patients (5/10) with recurrent disease, this was the second exposure to chemotherapy after receiving it at primary diagnosis. All were referred to RT following local tumor excision, of which 2/20 refused.

Twelve (75%) of the patients with LRR in the BCT group had mastectomy following LRR. Additional three patients in the subgroup of lumpectomy without RT followed by mastectomy (because of positive genetic testing), had local tumor excision. One patient with axillary recurrence had axillary lymph node dissection followed by axillary RT. One patient had partial breast re-irradiation. Eight (50%) of the patients in this group received chemotherapy following recurrence, all but one after exposure to chemotherapy at primary diagnosis.

### Distant recurrence

Distant recurrence as *first* failure was higher in the PMRT group (13.8%), with a median time to recurrence of 43 months (range 12–89), none having LRR preceding metastatic disease. In the non-PMRT cohort, only one patient (0.7%) had distant recurrence as the *first* recurrence event, and 20% (4/20) of patients with LRR had later metastatic recurrence at 20–37 months following LRR. In the BCT cohort, 9% (22/244 patients) had distant recurrence as *first* recurrence, and 19% (3/16) had later metastatic recurrence at 15–28 months following LRR.

### Overall survival

**Fig. 2:**
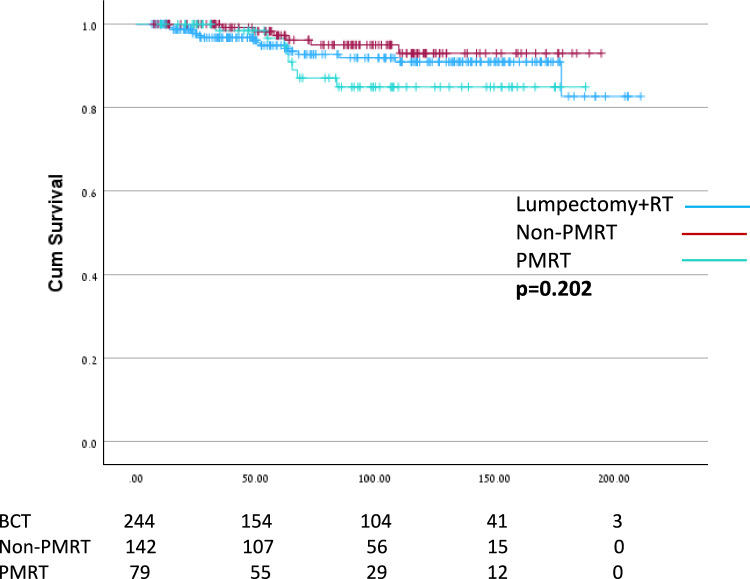
Overall survival. *PMRT* post-mastectomy radiation therapy

No significant difference in overall survival was observed between the study groups (Fig. 2).

## Discussion

The current study confirms the results of our previous report, demonstrating a higher incidence of LRR in *BRCA1/2* PV carriers with BC undergoing mastectomy without PMRT compared to patients treated with PMRT, despite the PMRT cohort having more advanced BC stage at diagnosis [15]. Consistent with previous observations, the non-PMRT cohort exhibited significantly elevated early LRR rates relative to the BCT cohort (14.7% vs. 5.1% at 5 years). Unlike our previous findings, this larger cohort with longer follow-up revealed a higher later cumulative LRR rate in the BCT group, with a median time to LRR of 69.9 months, compared to 17.5 months in the non-PMRT group. This may be explained by the protective effect of RT in the BCS group in the first few years after surgery, which is absent in the non-PMRT patients. These differences were numerically notable at the follow-up of 15 years (35% in BCT versus 16.6% in non-PMRT), but not found to be statistically significant, likely due to the small number of patients at risk at longer follow-up. Another possible explanation for the statistically non-significant difference in LRR rates could be the higher rate of competing events, especially CBC treated with systemic therapy and metastatic disease in the BCT group.

In our previous study, we assumed that residual subclinical disease was the main factor driving the high rate and early occurrence of local recurrences in the non-PMRT group [15]. Limited by the small number of patients and events at a longer follow up, our current analysis supports that assumption. In contrast, the late increase of recurrences after BCT suggests the emergence of second primary tumors rather than true local recurrences [16].

Notably, despite more advanced disease, we observed only one event of LRR in the PMRT group at a median follow-up of nearly 7 years. This group most probably had similar amounts of residual breast tissue and larger amounts of residual tumor cells (thereby the indication for PMRT). This suggests the importance of radiation therapy in treating residual disease in the breast tissue after SSM/NSM [6]. As expected, disease stage is the most important factor associated with the risk for distant failure, and the PMRT group had a higher incidence of metastatic recurrence compared to the non-PMRT cohort.

Before diagnosis, two-thirds of non-PMRT patients were aware of their genetic status and actively monitored at high-risk clinics, which may explain the earlier stage at diagnosis [17], and the choice of contralateral RRM in 95% of this cohort at the time of the primary surgery. However, the surgical procedure of NSM/SSM without RT did not provide sufficient local control for these patients.

Moreover, patients with genetic risks are often encouraged to have RRM. However, an increasing number of patients who are eligible for BCT without hereditary predisposition syndromes chose to undergo unilateral or bilateral mastectomy with immediate breast reconstruction [18]. This trend is most probably driven by the fear of additional cancer, a desire to avoid RT in the setting of breast conservation, the wish for symmetry with the therapeutic mastectomy, and the sociocultural environment including the organization of the health care system. Therefore, the oncological safety of SSM/NSM should be re-evaluated for non-*BRCA1/2* PV carriers regarding superficial margins, residual tumor foci within the skin flap, and residual breast tissue. Only 1/204 patients (0.5%) who underwent RRM had a CBC in our study, but longer follow-up is necessary to assess the risk of developing breast cancer in the residual breast tissue after RRM, which is expected to be significantly lower than the annual 1–2% risk for breast cancer in the *BRCA1/2* PV population without RRM [19].

Despite an increased LRR rate in the non-PMRT cohort, there were no significant differences in estimated overall survival rates among the study groups. This finding aligns with previous studies showing no differences in outcomes in *BRCA1/2* PV carriers regardless of loco-regional therapy [6–12], potentially due to the role of salvage treatments after LRR or CBC. In our cohort, the diagnosis of LRR led to additional chemotherapy in half of the patients, along with its associated morbidity. Most of them had RT, which was deemed unnecessary after the primary surgery. Of those with LRR, 20% developed metastatic disease and subsequently died. Although a direct relationship between local recurrence and metastatic disease cannot be established in these cases, this further emphasizes the importance of preventing LRR. These outcomes should be discussed with patients before treatment decisions, informing them about the likelihood of additional cancer and potential oncological treatments, even though these factors might not significantly impact overall survival.

There are several inherent limitations to our study. This is a single-institution retrospective study, with a limited number of participants and a narrow spectrum of *BRCA1/2* PVs predominant in the Israeli population [20]. We did not analyze the rate of LRR according to the different types of mastectomy (e.g., NSM versus SSM, prepectoral versus retropectoral implants). Due to insufficient documentation, some pathological variables, such as tumor grade and lymphovascular space invasion, could not be evaluated as recurrence risk factors. Lastly, the cohort's prevalent nature may have led to survivorship and ascertainment biases. However, the uniqueness of this study lies in its approach: previously published studies [8–12] collectively compared patients undergoing mastectomy irrespective of PMRT administration to those undergoing BCT, and some of them lack data on the proportion of SSM/NSM cases. Consequently, they could not assess the effect of PMRT avoidance on LRR, especially with newer operation techniques.

We do not support PMRT in all patients, as it is associated with higher complication rates, particularly in cases of implant-based reconstruction [21]. However, we do plea for further multidisciplinary research to improve the outcomes after RRM.

## Data Availability

The data underlying this article cannot be shared publicly due to the privacy of individuals that participated in the study. The data will be shared on reasonable request to the corresponding author.
